# Factors Affecting the Interval Between Marriage and the Birth of the First Child 2018–2023: A Retrospective Cohort Study

**DOI:** 10.1002/hsr2.73037

**Published:** 2026-08-16

**Authors:** Mohsen Adak, Arian Azadnia, Mohammad Aziz Rasouli, Ghobad Moradi, Bijan Nouri

**Affiliations:** ^1^ Student Research Committee Kurdistan University of Medical Sciences Sanandaj Iran; ^2^ Health Metric and Evaluation Research Center, Research Institute for Health Development Kurdistan University of Medical Sciences Sanandaj Iran; ^3^ Social Determinant of Health Research Center, Research Institute for Health Development Kurdistan University of Medical Sciences Sanandaj Iran; ^4^ Center for Communicable Disease Control Iran Ministry of Health and Medical Education Tehran Iran

**Keywords:** cohort study, Cox proportional hazards model, first birth interval, Iran

## Abstract

**Background and Aim:**

The interval between first births directly affects family size and maternal and child mortality. The aim of this study was to investigate the factors associated with the timing of first birth after first marriage among women in Sanandaj city.

**Methods:**

In this retrospective cohort study, data from 1200 women aged 15 to 49 years were collected in Sanandaj city (western Iran) in 21 March 2018–2023. Multistage random sampling method was used in this study. The study population was divided into three categories of urban, urban‐rural, and rural health centers. Data on the study subjects were collected for the final study between October and December 2024. Multivariate Cox regression was used to evaluate predictors of birth interval to marriage and calculate adjusted hazard ratios.

**Results:**

In a study of 1200 subjects, 720 number of censored (60%), the median interval between the birth of the first child after marriage was 3.4 years (3.2–3.5). The multivariate Cox regression analysis revealed that each additional year of age at marriage was associated with a 7% increase in the hazard of having a shorter interval to first birth (HR = 1.07, 95% CI: 1.04–1.09, *p* = 0.001). The multivariate analysis also showed that a 1‐year increase in age at menarche was associated with a 14% higher hazard of a shorter marriage‐to‐first‐birth interval (HR = 1.14, 95% CI: 1.05–1.24, *p* = 0.001). Furthermore, women exposed to government incentive policies had a 45% higher hazard of experiencing a shorter first birth interval (HR = 1.45, 95% CI: 1.17–1.79, *p* = 0.001).

**Conclusion:**

Increasing age, increasing age of menarche, and government incentive policies are the most effective predictors of a short interval between births after marriage. The government's attention to incentive policies and their support can lead to a decrease in the interval between births after marriage.

## 1 Introduction

The interval between marriage and the birth of the first child, commonly referred to as the ‘first birth interval,’ is a critical indicator in fertility epidemiology and the analysis of population dynamics. This interval not only influences family size and total fertility rates but also has implications for maternal and child health outcomes [1]. While adverse maternal and neonatal outcomes such as preterm birth, low birth weight, and maternal depletion syndrome are primarily associated with short intervals between consecutive births (interpregnancy intervals) where the mother's body has not fully recovered, the first‐birth interval is particularly important as a marker of fertility timing and a key determinant of completed family size [1, 2]. A shorter first‐birth interval may influence subsequent fertility patterns by extending the reproductive window and reducing age‐related subfertility risks. Studies have demonstrated that early first birth is associated with higher completed parity in populations with strong preferences for larger families, while delayed first birth is consistently linked to lower total fertility [1, 2]. Numerous studies have explored the factors affecting the first birth interval; however, many have overlooked the presence of women who, for various reasons including infertility, do not give birth. This limitation can impact the accuracy of results and underscores the need for more advanced statistical models [3]. In Iran, the epidemiology of the first birth interval has been shaped by recent demographic and social transformations. The average interval from marriage to first birth in the country is reported to be approximately 2.7 years, though this varies across regions [4]. For instance, in rural areas, this interval is typically shorter than in urban areas, attributed to cultural factors, limited access to family planning services, and lower education levels [5]. The decline in the total fertility rate from 6.9 births per woman in 1950 to 1.9 in 2005, coupled with its continued downward trend in recent years and the implementation of pro‐natalist incentive policies since 2014, has highlighted the need for a more detailed examination of this interval at the regional level [6, 7].

It is important to note, however, that in demographic analysis, a distinction exists between fertility timing (tempo) and completed family size (quantum). Accelerating the transition to first birth does not automatically guarantee a larger final family size, as women may simply shift their childbearing earlier without altering their total number of children. Nevertheless, several pathways link earlier first birth to potentially higher fertility: a longer remaining reproductive lifespan provides more opportunities for subsequent births [6, 8]; earlier initiation reduces the risk of age‐related subfertility, which increases with delayed childbearing [4]; and earlier completion of childbearing reduces the risk of complications that could limit subsequent pregnancies [2]. In the Iranian context, where pro‐natalist policies since 2014 have explicitly targeted both the tempo and quantum of fertility [6, 7], understanding the determinants of the first‐birth interval is essential for evaluating these policy interventions. In Sanandaj, located in western Iran with its ethnic diversity and unique social characteristics, this topic offers valuable insights for health policy development.

Several factors influence the interval from marriage to the first birth, including age at marriage, educational levels of couples, economic status, access to contraception methods, and cultural norms [9]. Studies have shown that marriage at younger ages is often associated with a shorter interval to the first birth, while higher education levels among women tend to lengthen this interval, possibly due to delays in childbearing to pursue educational and career goals [10]. Additionally, government incentive policies, such as financial support or maternity leave, can shorten the first birth interval by reducing economic barriers [11]. Conversely, limited access to family planning services or traditional beliefs promoting early childbearing may decrease this interval [5].

The aim of this study is to investigate the factors influencing the first birth interval among women in Sanandaj city using the Cox proportional hazards model. Conducted as a retrospective cohort study, this research seeks to identify key determinants of the first birth interval and provide scientific evidence to enhance reproductive health programs and support population rejuvenation policies in Iran.

## 2 Methods

### 2.1 Study Setting, Study Population and Study Period

This retrospective cohort study was performed in Sanandaj, western Iran. The study population consisted of all women residing in Sanandaj who married between 21 March 2018 and 20 March 2023 and whose data were recorded in the local health centers. Data collection took place between October and December 2024.

### 2.2 Source of Data and Data Collection Procedures

Data for this study were collected using a checklist designed. Based on the study by Dehesh et al. in Kerman (Iran) [1], the hazard ratio for the variable of age at marriage was considered to be 1.3. Considering the power of the test of 90% and the confidence level of 95%, the required sample size was 1200 people. In this study, a multistage random sampling method was used. The study population was divided into three categories: urban, urban‐rural, and rural health centers. These categories included a number of health centers as clusters. There were 17 centers in the urban category, eight centers in the rural category, and five health centers in the urban‐rural category. Sampling was carried out in a two‐stage cluster; seven centers were selected from the urban category, three centers from the rural category, and two centers from the urban‐rural category. From each of the 12 selected centers, 100 people were randomly selected, for a total of 1200 people as the final sample. To select people in each year of the study, 10 people were selected from each center in each year by simple random sampling.

Data were collected using a structured checklist developed by the research team based on the study objectives. After explaining the study objectives and obtaining verbal consent, trained interviewers completed the checklist through face‐to‐face interviews with the women. The checklist included demographic variables (woman's and husband's, woman's age at menarche, education level), economic factors (woman's and husband's occupation, household income level), and individual and behavioral factors (smoking status, alcohol consumption, body mass index, contraceptive methods, and presence of underlying diseases such as hypertension, diabetes, and kidney disease). Defining all variables, including how they were measured/categorized shown in Supplementary file, Table S1.

Exposure to government incentive policies was assessed by asking participants whether they had received any pro‐natalist supports (including marriage loans, childbirth subsidies, maternity benefits, or housing incentives) between marriage and the birth of their first child. For participants who were difficult to reach, information on household income and selected variables was obtained via telephone interview with the woman or a first‐degree family member.

### 2.3 Ethical Approval and Consent to Participate

Ethical approval for this study was granted by the Ethics Committee of Kurdistan University of Medical Sciences (IR.MUK.REC.1402.074), and the research was conducted in accordance with the tenets of the Declaration of Helsinki.

### 2.4 Data Analysis

Descriptive statistics were summarized using frequency tables and means with standard deviations (SD) for categorical and continuous variables, respectively. The interval from marriage to the first birth (survival time) was analyzed for individuals across the examined variables. The outcome variable, first live birth, was considered censored for some participants who did not experience it by the study's end. The median survival time was estimated overall and by the examined variables. Analyses within different study subgroups were conducted using Kaplan‐Meier plots. The proportional hazards assumption was tested using graphical methods, including Schoenfeld residuals and cumulative hazard plots. Subsequently, the association of each variable with the time from marriage to the first birth was assessed using the univariate Cox proportional hazards model. Variables with a *p*‐value < 0.20 in the univariate Cox regression analysis were considered as potential candidates for inclusion in the multivariate model. This liberal p‐value threshold (*p* < 0.20) was used to avoid excluding potentially important variables that might act as confounders. Hazard ratios (HR) with 95% confidence intervals were estimated for all variables. Data analysis was performed using STATA 17 software, with a significance level set at *p* < 0.05.

## 3 Results

**Figure 1:**
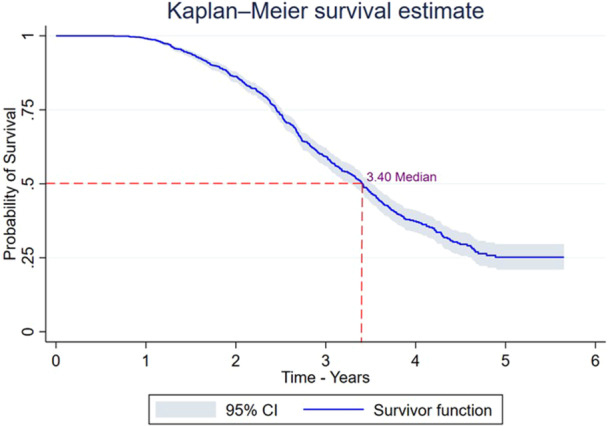
Kaplan‐Meier plot of the interval from marriage to the first birth.

The initial evaluation included a total of 1,200 women. During the study period, 720 participants (60%) were censored and 480 event (40%), and analyses were conducted on all 1,200 participants using survival analysis. As shown in Table 1, the mean (± SD) age of the participants was 29.18 ± 7.13 years, and the mean age at menarche (± SD) was 14.12 ± 1.58 years. Regarding demographic characteristics, 922 participants (76.9%) lived in urban areas and 278 (23.1%) in rural areas (Table 1). The majority of women had a university degree (527, 43.9%), followed by high school education (377, 31.4%). With respect to occupation, 895 women (74.6%) were housewives, while the majority of their husbands were employees (849, 70.8%). More than half of the households reported moderate income (645, 53.8%). Smoking was reported by 99 women (8.3%), and alcohol consumption by 194 women (16.2%). Supplementary health insurance coverage was present in 142 participants (11.8%), and 88 women (7.3%) had underlying diseases. The majority of women had normal BMI (555, 46.3%) or were overweight (456, 38.0%). Use of contraceptives was reported by 753 participants (62.8%). Regarding government incentive policies, 297 women (61.8%) reported receiving at least one type of incentive. Detailed descriptive statistics of the studied variables are presented in Table 1. The median interval between marriage and the birth of the first child was 3.40 years (Figure 1).

**Table 1:** Demographic distribution the birth of the first child after marriage (*N* = 1200).

Variable		*N* = 1200
Age (Mean ± SD)		29.18 ± 7.13
Menarche age (Mean ± SD)		14.12 ± 1.58
		*N* (%)
Place of residence	City	922 (76.9)
	Rural	278 (23.1)
Education	Elementary	80 (6.7)
	Middle school	216 (18)
	High school	377 (31.4)
	University degree	527 (43.9)
Job/Woman	Freelance	108 (9)
	Employee	196 (16.4)
	Housewife	895 (74.6)
Job/Man	Other (Unemployed, temporary job, etc.)	278 (23.2)
	Employee	849 (70.8)
	Worker	19 (1.8)
	Freelance	54 (4.5)
Income	Low	295 (24.6)
	Moderate	645 (53.8)
	High	260 (21.7)
Shiftwork/Man	Nightly	22 (1.8)
	Daily	1178 (98.2)
Shiftwork/Woman	Nightly	147 (12.3)
	Daily	1053 (87.7)
Smoking	No	1101 (91.7)
	Yes	99 (8.3)
Alcohol Use	No	1006 (88.3)
	Yes	194 (16.2)
Supplementary insurance	No	1058 (88.2)
	Yes	142 (11.8)
Underlying disease	No	1112 (92.7)
	Yes	88 (7.3)
BMI	Underweight (<18.5)	94 (7.8)
	Normal (18.5–24.9)	555 (46.3)
	Overweight (25–29.9)	456 (38)
	Obese (≥30)	95 (7.9)
Use of contraceptives	No	447 (37.2)
	Yes	753 (62.8)
Incentive policies^a^	No	183 (38.2)
	Yes	297 (61.8)

a Number of Incentive Policies = 480.

### 3.1 The Separate Survival Data Analysis

Before fitting the Cox‐PH model, the proportional hazards assumption was tested using a global test, all covariates have *p*‐values > 0.05, indicating no significant correlation between Schoenfeld residuals and survival time. The global test is also non‐significant (Chi‐Square = 15.26, *p*‐value = 0.29), confirming that the proportional hazards assumption is not violated (Supplementary File, Table S2). The separate survival analysis was conducted using the Cox proportional hazard model.

The multivariate Cox regression analysis showed that for each additional year of age at marriage, the hazard of a shorter interval to first birth increased by 7% (HR = 1.07, 95% CI: 1.04–1.09, *p* = 0.001) (Table 2).

**Table 2:** Factors affecting the birth of the first child after marriage using the Cox proportional hazards model.

		Cox univariate		Cox multivariate	
Variable		HR (%95 CI)	*p* value	HR (%95 CI)	*p* value
Age		1.02 (1.01–1.03)	0.001	1.07 (1.04–1.09)	0.001
Menarche age		1.12 (1.06–1.18)	0.001	1.14 (1.05–1.24)	0.001
Place of residence	City	Reference	—	Not in model^b^	—
	Rural	1.47 (0.91–1.83)	0.231	—	—
Education	Elementary	Reference	—	Reference	—
	Middle school	0.70 (0.46–1.08)	0.117	0.90 (0.57–1.41)	0.647
	High school	0.83 (0.56–1.23)	0.361	0.67 (0.44–1.02)	0.064
	Academic	1.44 (0.99–2.10)	0.052	0.81 (0.44–1.01)	0.055
Job/Woman	Freelance	Reference	—	Reference	—
	Employee	1.01 (0.70–1.42)	0.971	0.99 (0.68–1.44)	0.970
	Housewife	0.69 (0.51–0.95)	0.026	0.81 (0.73–1.03)	0.452
Job/Man	Other^a^	Reference	—	Not in model^b^	—
	Employee	1.23 (0.81–1.86)	0.318	—	—
	Worker	0.50 (0.19–1.30)	0.156	—	—
	Freelance	0.84 (0.57–1.25)	0.407	—	—
Income	Low	Reference	—	Reference	—
	Moderate	1.05 (0.84–1.31)	0.961	0.98 (0.77–1.25)	0.855
	High	1.54 (1.19–2.01)	0.001	1.14 (0.83–1.57)	0.288
Shiftwork/Man	Nightly	Reference	—	Not in model^b^	—
	Daily	0.98 (0.93–1.32)	0.942	—	—
Shiftwork/Woman	Nightly	Reference	—	—	—
	Daily	0.61 (0.30–1.23)	0.268	—	—
Smoking	No	Reference	—	Reference	—
	Yes	0.76 (0.54–1.08)	0.135	0.99 (0.77–1.25)	0.855
Alcohol Use	No	Reference	—	Reference	—
	Yes	0.74 (0.57–0.99)	0.044	1.14 (0.83–1.57)	0.110
Supplementary insurance	No	Reference	—	Reference	—
	Yes	1.12 (0.83–1.52)	0.119	0.93 (0.60–1.44)	0.768
Underlying disease	No	Reference	—	Ref	—
	Yes	0.93 (0.67–1.31)	0.711	—	—
BMI	Underweight (<18.5)	Reference	—	Reference	—
	Normal (18.5–24.9)	2.09 (1.31–3.34)	0.002	0.92 (0.56–1.51)	0.836
	Overweight (25–29.9)	2.20 (1.37–3.53)	0.001	0.94 (0.57–1.54)	0.893
	Obese (≥30)	2.03 (1.17–3.50)	0.011	1.14 (0.63–2.05)	0.660
Use of contraceptives	No	Reference	—	Not in model^b^	—
	Yes	1.25 (0.78–1.87)	0.231	—	—
Incentive policies	No	Reference	—	Reference	—
	Yes	1.20 (0.99–1.47)	0.057	1.45 (1.17–1.79)	0.001

a Other (Unemployed, temporary job, etc.).

b Not in model: *p*‐Value < 0.20 Note in multivariate Cox model.

In the multivariate analysis, each 1‐year increase in age at menarche was associated with a 14% higher hazard of a shorter marriage‐to‐first‐birth interval (HR = 1.14, 95% CI: 1.05–1.24, *p* = 0.001). Place of residence had no significant effect on the interval (HR = 1.47, 95% CI: 0.91–1.83, *p* = 0.231). Education level also showed no significant association with the interval in either univariate or multivariate analyses (*p *> 0.05) (Table 2).

In the univariate analysis, housewife women had a significantly lower hazard of a shorter interval compared to employed women (HR = 0.69, 95% CI: 0.51–0.95, *p* = 0.026); however, this association lost significance in the multivariate model (HR = 0.81, 95% CI: 0.73–1.03, *p* = 0.452). Husband's occupation was not significantly associated with the outcome (Table 2).

Higher household income was associated with a higher hazard of a shorter interval in the univariate analysis (HR = 1.54, 95% CI: 1.19–2.01, *p* = 0.001), but this effect was attenuated and became non‐significant in the multivariate model (HR = 1.14, 95% CI: 0.83–1.57, *p* = 0.288). Work shift (day vs. night) for both women and men did not significantly affect the interval. Smoking showed no significant association, while alcohol consumption was associated with a lower hazard of a shorter interval in the univariate analysis (HR = 0.74, 95% CI: 0.57–0.99, *p* = 0.044); however, this association was not significant in the multivariate model (HR = 1.14, 95% CI: 0.83–1.57, *p* = 0.110).

Underlying diseases had no significant effect on the interval. In the univariate analysis, women with normal (HR = 2.09, 95% CI: 1.31–3.34, *p* = 0.002), overweight (HR = 2.20, 95% CI: 1.37–3.53, *p* = 0.001), and obese BMI (HR = 2.03, 95% CI: 1.17–3.50, *p* = 0.011) categories showed higher hazards of a shorter interval compared to underweight women; however, these associations were not statistically significant in the multivariate analysis (Table 2).

Use of contraceptive methods was not significantly associated with the interval. In the multivariate Cox regression analysis, exposure to government incentive policies was independently associated with a significantly higher hazard of a shorter marriage‐to‐first‐birth interval (HR = 1.45, 95% CI: 1.17–1.79, *p* = 0.001) (Table 2).

## 4 Discussion

The results of the present study indicate that the median interval from marriage to the first birth was 3.4 years, with higher age at marriage, age at menarche, and government incentive policies identified as significant factors in reducing this interval. However, education, place of residence, occupation and work shifts for both women and men, monthly income, underlying diseases, smoking, alcohol consumption, and the use of contraception methods showed no significant impact on shortening the interval to the first birth after marriage.

The median first birth interval of 3.4 years reported in this study contrasts with the 7.3 years reported by Mahmoudi et al. in West Azerbaijan [3]. The current study showed that for each additional year of age at marriage, the hazard of a shorter interval to first birth increased. In contrast, a study by Dehesh involving 1,350 women identified the age of women and their husbands at marriage as the most influential factors on the first birth interval [1]. Miri et al.'s study demonstrated a significant effect of maternal age at marriage and place of residence on the interval to the first birth [12]. Nouri et al.'s research on 882 women in southern Iran highlighted age and regular menstruation as key determinants of pregnancy timing. Regarding sociodemographic variables, couples marrying at older ages were more likely to report a shorter interval to the first birth, a finding consistent with studies from Nigeria, Iran, and southern Iran [13, 14]. Additionally, a similar study in India reported a positive association between increasing age at marriage and a shorter first birth interval [15], which may be explained by the limited fertile window, particularly for women. Women marrying at older ages have a shorter reproductive lifespan, prompting an earlier first birth [16, 17].

The multivariate analysis revealed that a 1‐year increase in age at menarche was associated with a 14% higher hazard of a shorter first birth interval (HR = 1.14, 95% CI: 1.05–1.24). Results of Dehesh et al. study showed a significant association between the first birth interval and age at menarche [1], noting that women experiencing menarche at younger ages were more likely to report a longer interval to the first birth. This aligns with previous findings [18], possibly due to early‐married girls delaying pregnancy due to their young age at marriage or hormonal irregularities and menstrual disorders contributing to an extended interval. While this finding may initially seem counterintuitive, it carries biological plausibility. Early menarche is frequently accompanied by a longer phase of irregular ovulation and anovulatory cycles due to immaturity of the reproductive axis. Conversely, later menarche is often followed by quicker attainment of regular ovulatory function, resulting in higher fecundability. Furthermore, early menarche has been associated with increased risk of conditions such as polycystic ovary syndrome, hyperandrogenism, and insulin resistance, which may contribute to subfertility. In the cultural context of this study, women with later menarche may also enter marriage at older ages, prompting them to initiate childbearing sooner within a narrower reproductive timeframe [4, 19, 20].

Results of present study found no significant effect of education on first birth interval, while results of other studies have identified higher education levels among couples as a key predictor of a longer first birth interval, with educated individuals more likely to delay childbearing [17, 19, 20]. Other studies in India support education as a factor influencing the first birth interval [15]. Similarly, a Nigerian study suggested that higher education delays marriage and, with increasing maturity, may lead to more effective contraception, thus affecting the birth interval [13]. Education enhances job opportunities in modern sectors, competing with childbearing demands, and empowers women with income‐generating roles, influencing decisions on family size and birth spacing. Although higher education is typically associated with delayed childbearing, no significant association was observed in this study. This null effect is likely mediated by occupational status, as the majority of participants (74.6%) were housewives, and homemaker status was strongly correlated with lower education levels. This high proportion of homemakers is consistent with the prevailing traditional gender roles and limited employment opportunities for women in Sanandaj and Kurdistan province.

Although differences between urban and rural settings in fertility patterns were mentioned in the introduction, place of residence was not significantly associated with the interval between marriage and first birth in this study (HR = 1.47, 95% CI: 0.91–1.83, *p* = 0.231). This finding may reflect the ongoing convergence of reproductive behaviors between urban and rural areas in Iran resulting from improved access to education, healthcare services, family planning programs, and mass media, as well as significant rural‐to‐urban migration [1, 7, 12].

Studies also indicate that rural couples tend to have more children to assist with agricultural and other tasks, aligning with a preference for shorter birth intervals [12, 19, 21].

The present study's univariate Cox regression results identified body mass index (BMI) as a significant factor in reducing the first birth interval, though this effect was not significant in multivariate analysis after adjusting for confounders. Previous research [22] and findings from Kerman support a positive role of higher BMI in extending the first birth interval [1], potentially due to obesity's impact on fertility. Obesity, linked to polycystic ovary syndrome characterized by oligo‐ or anovulation, hyperandrogenism, menstrual irregularities, and infertility, may delay first births despite a desire for earlier childbearing [23, 24].

The univariate analysis in this study suggested that higher income reduced the first birth interval, though this effect was not significant in multivariate. Whereas studies in Kerman and rural Shiraz in Iran found that families with sufficient income delayed their first birth [1, 25], consistent with a Bangladesh study linking higher economic status to longer birth intervals [21].

The loss of statistical significance for several variables (e.g., household income, housewife status, BMI, and alcohol consumption) in the multivariate analysis likely reflects confounding and mediation effects. Higher‐income women and housewives tended to marry at older ages and had greater exposure to incentive policies. Once age at marriage and incentive policies were adjusted for, the independent effects of these socio‐economic and behavioral variables were substantially attenuated. This pattern is common in fertility research, where socio‐economic and behavioral factors are highly interrelated and often mediated through key demographic variables such as age at marriage. The final parsimonious model highlights the importance of multivariable adjustment to identify true independent predictors.

The current study found no significant effect of contraception use on reducing the first birth interval, aligning with an Indian study [26], though Chernet's study in Ethiopia and a Nigerian study reported that contraception use increased this interval [10, 27]. Previous research also highlights a strong association between contraception and birth spacing [27, 28], with cultural and economic differences in healthcare access and restrictive policies, particularly in rural areas with limited contraception availability, potentially influencing birth interval variations in Iran.

In the present study, neither smoking nor alcohol consumption during the marriage‐to‐first‐birth interval showed a significant independent association with the outcome in the multivariate analysis. This may be partly due to the relatively low prevalence of these behaviors in the study population (8.3% for smoking and 16.2% for alcohol), which is consistent with cultural and religious norms in Iran that generally discourage alcohol consumption and smoking among women of reproductive age.

The independent association between government incentive policies and a shorter first birth interval (HR = 1.45, 95% CI: 1.17–1.79) indicates that pro‐natalist measures can effectively influence reproductive timing. To maximize impact, policymakers should focus on multi‐component packages rather than isolated interventions. The most effective policies are likely to include: direct financial support (marriage loans, childbirth subsidies, and housing grants), extended paid parental leave (minimum 9–12 months) with job protection, affordable, high‐quality childcare services, and targeted assistance for low‐ and middle‐income families as well as highly educated women. International evidence from France, Norway, and South Korea shows that combining financial incentives with accessible childcare and family‐friendly workplace policies produces more sustained effects on fertility than financial support alone. In Iran, expanding existing marriage and housing loan programs while adding quality childcare and parental leave benefits could more effectively reduce the interval to first birth and support population policy goals [29, 30, 31].

Policymakers should prioritize multi‐component packages including marriage and housing loans, extended paid parental leave, and subsidized childcare, with special attention to low‐ and middle‐income as well as highly educated couples [32, 33]. While such policies require substantial investment, they can be fiscally sustainable and cost‐effective over time by mitigating the economic consequences of population aging [34]. Phased implementation, targeting, and integration with existing programs would enhance feasibility in the Iranian setting.

## 5 Conclusion

The findings of this study indicate that increasing age at marriage, age at menarche, and government incentive policies are the most effective predictors of a shorter first birth interval. Government attention to incentive policies and their support could contribute to reducing the interval from marriage to the first birth.

### 5.1 Limitations of the Study

This study has several limitations that should be considered when interpreting the findings. Most variables, including BMI, household income, alcohol consumption, smoking, and exposure to incentive policies, were collected through self‐report. Self‐reported data are susceptible to recall bias, social desirability bias, and misclassification. For example, BMI calculated from self‐reported height and weight tends to underestimate true obesity, while income may be under‐ or over‐reported depending on participants' socioeconomic perceptions. Similarly, self‐reported alcohol consumption and exposure to government incentive policies may be subject to under‐reporting due to cultural or social stigma. Although we attempted to minimize these biases through structured interviews and verification where possible, the potential for measurement error remains. These limitations may have led to some attenuation of associations or residual confounding in the multivariate models.

Despite these limitations, the use of survival analysis for a time‐to‐event outcome and the consistency of key findings (particularly age at marriage and incentive policies) with existing literature strengthen the credibility of the main results.

## Author Contributions

**Mohsen Adak:** conceptualization, methodology, data curation, investigation, writing – review and editing, writing – original draft, software, formal analysis. **Arian Azadnia:** conceptualization, investigation, validation, methodology, software, formal analysis, writing – original draft, writing – review and editing. **Mohammad Aziz Rasouli:** methodology, data curation, visualization, writing – original draft, writing – review and editing. **Ghobad Moradi:** conceptualization, methodology, investigation, writing – original draft, writing – review and editing, validation, data curation, software, formal analysis. **Bijan Nouri:** conceptualization, methodology, writing – review and editing, writing – original draft, supervision, data curation, software, investigation, validation, formal analysis.

## Funding

The authors have nothing to report.

## Conflicts of Interest

The authors declare no conflicts of interest.

## Transparency Statement

The lead author Bijan Nouri affirms that this manuscript is an honest, accurate, and transparent account of the study being reported; that no important aspects of the study have been omitted; and that any discrepancies from the study as originally planned have been explained.

## Supporting information


Supporting File

## Data Availability

The data that support the findings of this study are available from the corresponding author upon reasonable request. The data that support the findings of this study are available on request from the corresponding author. The data are not publicly available due to privacy or ethical restrictions. The datasets generated and/or analyzed during the current study are available from the corresponding author upon reasonable request.
