# Supplementary material for: Factors Affecting the Interval Between Marriage and the Birth of the First Child 2018–2023: A Retrospective Cohort Study
Source: Health Sci Rep. 2026 Aug 16;9(8):e73037. doi: 10.1002/hsr2.73037 (PMC13478848; doi:10.1002/hsr2.73037)
Supplement: Supplementary file 1 [file HSR2-9-e73037-s001.docx]

**Table S1.** Defining all variables, including how they were measured/categorized

| Variable | Definition / Categorization | Measurement Method/ Structured face-to-face interviews |
| --- | --- | --- |
| Age at marriage | Age in completed years at the time of marriage | Self-report (continuous) |
| Age at menarche | Age in completed years at first menstruation | Self-report (continuous) |
| Place of residence | Urban / Rural | Based on health center registration |
| Education level | Elementary, Middle school, High school, University degree | Highest completed level (categorical) |
| Job/ Woman | Housewife / Employee / Freelance | Self-report during the interval |
| Job/ Man | Employee / Worker / Freelance / Other ((Unemployed, temporary job, etc.) | Self-report |
| Household income | Low / Moderate / High (based on local criteria) | Self-report (monthly income) |
| Shift work (Woman/Man) | Day shift / Night shift | During the marriage-to-first birth interval |
| Smoking | Yes / No (any tobacco use) | During the interval (self-report) |
| Alcohol consumption | Yes / No | During the interval (self-report) |
| BMI | Underweight (<18.5), Normal (18.5–24.9), Overweight (25–29.9), Obese (≥30) | Calculated from height and weight measured |
| Use of contraceptives | Yes / No (any modern method) | During the interval |
| Incentive policies | Yes / No | Receipt of any government pro-natalist support (marriage loan, childbirth subsidy, maternity benefits, housing incentives, etc.) during the interval (self-report, verified when possible) |
| First birth interval | Time (in years) from marriage to first live birth | Survival time (censored if no birth by study end) |

**Table S2**. Cox proportional hazard assumption test

| Variable | rho | chi2 | df | Prob>chi2 |
| --- | --- | --- | --- | --- |
| Age | -0.05157 | 1.11 | 1 | 0.2913 |
| Menarche age | -0.00900 | 0.03 | 1 | 0.8645 |
| Place of residence | -0.00033 | 0.00 | 1 | 0.9950 |
| Education | -0.00943 | 0.03 | 1 | 0.8608 |
| Job/ Woman | -0.06297 | 1.29 | 1 | 0.2554 |
| Job/ Man | -0.01903 | 0.12 | 1 | 0.7275 |
| Shiftwork/ Woman | -0.00591 | 0.01 | 1 | 0.9133 |
| Shiftwork/Man | -0.02636 | 0.26 | 1 | 0.6113 |
| Income | -0.10902 | 3.47 | 1 | 0.0645 |
| Smoking | 0.04564 | 0.72 | 1 | 0.3957 |
| Alcohol use | -0.03432 | 0.41 | 1 | 0.5233 |
| Supplementary insurance | 0.03084 | 0.36 | 1 | 0.5485 |
| Underlying disease | 0.02660 | 0.26 | 1 | 0.6087 |
| BMI | -0.09026 | 2.97 | 1 | 0.0846 |
| Use of contraceptives | 0.01645 | 0.10 | 1 | 0.7474 |
| Incentive policies | 0.09713 | 3.26 | 1 | 0.0708 |
| Global test |  | **15.26** | **16** | **0.2974** |
